# From law enforcement to public safety: Police officer experiences of naloxone administration to reverse opioid overdose in New York City

**DOI:** 10.1371/journal.pone.0341062

**Published:** 2026-01-15

**Authors:** Bennett Allen, Alex Harocopos

**Affiliations:** 1 Department of Population Health, NYU Grossman School of Medicine, New York, New York, United States of America; 2 Bureau of Alcohol and Drug Use Prevention, Care, and Treatment, NYC Department of Health and Mental Hygiene, Queens, New York, United States of America; Virginia Commonwealth University School of Medicine, UNITED STATES OF AMERICA

## Abstract

The overdose epidemic has reshaped law enforcement’s relationship with public health, as police increasingly adopt overdose response measures, including naloxone training and use. This study analyzed 15 interviews with New York Police Department (NYPD) officers to examine their experiences administering naloxone in their duties. Naloxone was seen as facilitating a shift from traditional law enforcement to a broader public safety role, and officers noted its potential to improve public perceptions of police. However, tensions emerged as officers navigated dual roles in enforcement and health, with concerns that overdose reversal might enable continued drug use or crime. Additionally, officers expressed frustrations about naloxone’s limitations, particularly its inability to address systemic barriers to addiction recovery. These findings underscore the need for clear policies, comprehensive training, and stronger interagency partnerships to enhance the integration of public health strategies within policing and better support officers in responding to the overdose crisis.

## Introduction

Overdose remains a leading public health crisis in the United States (US). Despite substantial national declines from 2023–2024, approaching pre-pandemic levels [1], overdose remains a national epidemic, with over 70% of overdose deaths related to opioids [2]. As the leading cause of death among persons under age 50 in the US [3], overdose mortality has been accelerated through the introduction of illicitly manufactured fentanyl into the US drug supply [4,5]. The collateral consequences of the epidemic are profound: hepatitis C infections have increased [6,7], HIV has resurged through injection drug use-related outbreaks [8–10], and an estimated nearly 2 million children and adolescents have been placed in crisis due to parental or caregiver opioid use disorder (OUD) or overdose [11]. The annual economic toll of the epidemic is in excess of $1 trillion [12], including an estimated $14.8 billion annually in criminal justice costs [13]. National responses to the overdose epidemic are necessarily multi-modal, representing a unique integration of the public health, healthcare, social service, and criminal legal sectors [14].

The overdose crisis has placed extraordinary demands on law enforcement, who in many jurisdictions are the first responders to overdose-related emergencies [15]. In response, police officers have been equipped with naloxone, a safe medication that can reverse the effects of an opioid overdose [16]. The use of naloxone by police—once a strictly health intervention in the domain of emergency medical services—reflects a growing recognition by public safety and public health policymakers of the varied and dynamic roles that law enforcement can play in the overdose crises [17]. Local jurisdictions in all 50 states have equipped police with naloxone, suggestive of a paradigm shift in the role of police in drug enforcement [18] However, the integration of public health approaches to the overdose crisis by police raises crucial questions of how police officers perceive their evolving responsibilities, particularly in contexts where their actions intersect with public health and community engagement [19].

Prior research on law enforcement responses has documented law enforcement training in overdose response [20–23], police officer knowledge, perceptions, understanding, and implementation of overdose response laws (e.g., Good Samaritan Laws and syringe access laws) [24–28], officer training in and use of naloxone [29–32], and officer perceptions of people who use drugs (e.g., stigma toward people with OUD, knowledge and beliefs about OUD, and discretion to engage individuals with OUD in diversion and deflection) [33–38]. Considerable attention has been paid by researchers to the training of police officers in public health responses to overdose, and studies in this area have documented law enforcement officer attitudes toward overdose and overdose reversal [35,39], training in public health approaches to drug use [21,34], and substance use and people who use drugs [38,40,41]. Additional research has documented concerns about officer and department liability with respect to naloxone administration at overdose scenes [32]. However, less attention has been paid to the direct experiences of officers as they implement overdose response in the line of duty [29^,42].^ As more police departments across the US seek to further integrate public health practice and law enforcement through public health/public safety partnerships [43], how officers are responding is a pressing question.

In New York City (NYC), the site of this study, New York Police Department (NYPD) patrol officers were equipped with and trained to carry and administer naloxone in 2014 [44]. As in many US jurisdictions, NYPD officers are typically among the first to arrive at overdose scenes, a position that helped catalyze the department’s naloxone training policy [17]. As the largest local law enforcement agency in the US, NYPD’s actions exert tremendous national influence with respect to other jurisdictions’ adoption of policing policies and practices [45]. Likewise, initial implementation of the NYPD naloxone programs formalized a blurring of role boundaries between emergency medical and police personnel within NYC’s intergovernmental ecosystem, highlighting the need for cross-sector coordination and clarity in overdose response roles [17]. As such, it is crucial for researchers to attend not only to the systemwide adoption and implementation of public health approaches to policing, but also to the individual experiences of the field officers as they put these policies into practice, with the NYPD offering a unique and optimal setting to probe these experiences.

At the time of this study, NYPD officers’ overdose response occurred within a New York City and State policy environment that was already oriented toward encouraging emergency help-seeking and expanding community access to naloxone. Passed in 2011, New York State’s Good Samaritan protections were intended to reduce fear of calling 911 during an overdose by providing limited legal protections for witnesses and victims of overdose events [46]. In parallel, naloxone access was being expanded beyond clinical settings through registered community distribution and pharmacy access pathways, such that officers often arrived to scenes in which bystanders or family members might also possess naloxone or have already attempted reversal [47]. These local conditions are important for interpreting officers’ accounts of role ambiguity, scene dynamics, and perceived opportunities and limits for effectively responding to overdose.

This study explores the experiences of NYPD officers who administered naloxone in overdose situations in the line of duty in NYC. Drawing on qualitative data from interviews with 15 officers, our findings contribute to the literature on police involvement in public health interventions, with implications for law enforcement policy, training, and the continued evolution of integrated public health and public safety approaches to overdose response. Our study offers a time-specific snapshot that helps characterize enduring role tensions and implementation challenges that remain salient as overdose response strategies continue to develop. By documenting officers’ perspectives on their direct experiences of naloxone administration at a time when such experiences were particularly novel, this study sheds light on the role of police within the overdose epidemic, suggesting avenues for improving both officer support and community outcomes in overdose prevention.

## Methods

Between 16 June 2016 and 08 July 2017, we conducted in-depth, semi-structured interviews with 39 individuals aged 18 and older who had administered naloxone to reverse an opioid overdose in the 24 months prior to interview. All were residents of New York City or Long Island, New York, and belonged to one of three distinct social roles: NYPD officers (n = 15); family members or friends of persons who use opioids, but did not themselves use opioids (n = 9); and, persons who use opioids (n = 15) [48]. This analysis includes the sub-sample of NYPD officers (n = 15), recruited between 30 November 2016 and 15 March 2017.

A convenience sample of NYPD officers was recruited with the support of precinct-level leadership across NYC, who were informed of the study through NYPD Central Command channels. Precinct leadership identified patrol officers from their respective precincts who had administered naloxone in the past 24 months in the course of their official NYPD duties and referred them to the study. All participants were active NYPD officers at the time of both naloxone administration and interview. Notably, the sample purposefully included officers who had personally administered naloxone as part of their NYPD duties to ensure that interviewees could speak from direct experience rather than speculation, thus generating data on how police officers navigate overdose reversal experiences in real-world practice. Officers who had used naloxone were better positioned to reflect on moment-to-moment decision-making, perceived role conflict, and emotional or ethical responses that might not be captured in more general attitudinal research inclusive of officers who had not personally administered naloxone.

Prior to each interview, participants received a written informed consent procedure, including a detailed review of the study protocol, the risks and benefits of participation, and the right to terminate the interview at any time. The interview guide was semi-structured, with broad categories including the: relationships between the police and people who use drugs; police roles in overdose response; additional duty and labor associated with naloxone preparedness and use; naloxone training received; experiences administering naloxone; PWUD reaction to naloxone administration; impact of naloxone administration experiences on officers; and treatment of PWUD after naloxone administration. The semi-structured interview guide was developed based on a review of emerging literature on police overdose response and harm reduction practices at the time, as well as the study team’s prior experience conducting qualitative research in criminal legal and public health settings. While police officers were not directly involved in the development of the guide, the questions were informed by themes identified in early studies of naloxone implementation in law enforcement contexts, including issues of training, public perception, role conflict, and structural barriers to care. The guide was designed to elicit both descriptive accounts of naloxone administration and officers’ perspectives on their evolving professional responsibilities. Questions were open-ended, and participants were encouraged to elaborate on their experiences.

Interviews, ranging from 13 to 54 minutes in duration (mean = 31 minutes), were conducted in private office space at the NYPD Academy in Queens, NY, audio recorded, and professionally transcribed for analysis. Officers were not compensated for their participation and are identified here only by unique study code. All study methods and procedures were approved by the NYC Department of Health and Mental Hygiene Institutional Review Board (Protocol #16–029). Findings are reported in accordance with COREQ guidelines [49].

Transcripts were analyzed using Dedoose data analysis software. Utilizing a thematic approach to analysis [50], transcripts were read, re-read, and tentatively classified to identify patterns and relationships relating to the research questions. Following this, a list of initial codes was produced via line-by-line coding, which were then grouped into categories and combined to form a hierarchy of themes and subthemes. In the preliminary stages, a sample of transcripts were coded by two members of the research team and coding differences were discussed and reconciled until codes were agreed upon. Analytic memos were used to record codes and their definitions, and to think through the data [51]. Finally, categories were refined to produce a coherent thematic landscape. Throughout the process, regular team meetings were held to discuss emergent themes and reconcile discrepancies in coding and interpretation.

Across themes, perspectives varied in how broadly they were shared. Some themes reflected views voiced by many officers across interviews, whereas others reflected views expressed by a smaller subset of participants. In several interviews, officers articulated internally conflicting views, underscoring the ambivalence of occupying roles that bridge enforcement and care. To enhance transparency about thematic breadth without formal quantification, we report findings using conventional qualitative descriptors (e.g., “most,” “many,” “several,” “a few”) and highlight within-participant ambivalence and negative cases where they emerged [52].

### Researcher positionality

This study was conducted by researchers trained in public health and qualitative methods, with varying degrees of prior experience engaging law enforcement and harm reduction systems. None of the researchers were current or former police officers, and most approached this project from a perspective grounded in public health. These orientations shaped the study’s design, including the decision to center officer perspectives while also critically examining tensions between enforcement and care [53]. We acknowledge that our outsider status to law enforcement may have influenced how officers responded in interviews [54], such as potentially downplaying controversial views or emphasizing public health alignment. Furthermore, in interpreting the data, our values and prior experiences likely shaped theme development, particularly regarding issues of stigma, institutional roles, and systemic fragmentation. We sought to mitigate any potential biases through our conduct of analytic memos and team-based coding, as well as critical discussion of divergent interpretations, but we recognize that our positionality remains embedded in the analytic process. Future studies on the police role in overdose prevention may benefit from participatory methods or researcher-practitioner collaborations to more fully account for these dynamics [55].

## Results

### Participant characteristics

A total of 15 NYPD officers participated in this study (Table 1). The median age of officers was 35 years (range 25–49 years). Four (27%) identified as female, and 11 (73%) identified as male. Three (20%) of participants identified their ethnicity as Hispanic/Latino, and nearly all (N = 13; 87%) participants identified their race as White/Caucasian. Educational attainment varied, with 3 participants (20%) holding an associate’s degree, 4 participants (27%) reporting some college completion, 6 participants (40%) holding a bachelor’s degree, and 2 participants (13%) holding a postgraduate degree. All participants (N = 15; 100%) identified their sexual orientation as straight/heterosexual. The median number of times officers had administered naloxone prior to interviews was 2 (range 1–12).

**Table 1:** NYPD officer characteristics.

*Characteristic*	N (%)
Total	15 (100)
Age (years)	
Median (range)	35 (25-49)
Gender	
Male	11 (73)
Female	4 (27)
Race	
White/Caucasian	13 (87)
Hispanic/Latino	1 (7)
Multi-Racial	9 (7)
Ethnicity	
Hispanic/Latino	3 (20)
Not Hispanic/Latino	12 (80)
Sexual Orientation	
Straight/Heterosexual	15 (100)
Number of naloxone administrations	
Median (range)	2 (1-12)

*Source*: Authors’ analysis of data from interviews with NYPD officers

*Notes*: Percentages may not sum to 100 due to rounding

### Emergent themes

Five major themes emerged from our analysis of NYPD officers’ experiences administering naloxone in the course of their duties:

- *Transitioning officer roles from law enforcement to public safety*: A shift in officer identity and daily responsibilities, with naloxone use as part of a broader public safety mission
- *Naloxone as a tool to soften public perceptions of police*: Use of naloxone as a way to build trust and improve police-community relations by showing care for PWUD
- *Overdose reversal as facilitating drug use and crime*: Concerns that frequent reversal might encourage continued drug use or embolden criminal behavior
- *People who use drugs as unworthy of revival*: Moral judgments or fatalistic views about PWUD, sometimes questioning the value of repeated overdose reversals
- *Naloxone as a limited tool in the face of cross-system failures*: Broader systemic breakdowns in housing, treatment, and mental health services that naloxone alone could not address

Each theme is explored in detail below, supported by officer quotations that illustrate the complexity of their perspectives.

### Transitioning officer roles from law enforcement to public safety

Participants described their enhanced role to respond to overdoses as facilitating a shift toward the broader remit of public safety rather than focusing on law enforcement alone. In addition to their existing enforcement-oriented equipment, carrying naloxone meant that officers now had a tool which enabled them routinely to save lives, described by one participant as complementary to their existing responsibilities.

“As a police officer you have some of the highest responsibilities. I mean, you could be using force at any time. So, it’s good to have another responsibility that’s not taking life, but giving life. It’s nice.”

*(Participant ATR02)*

For some participants, this transition facilitated the opportunity to help people in a different, more affirmative way. The routine work of patrol officers is taxing and often involves negative interactions with individuals and communities, which some officers contrasted to the positive, life-saving work of emergency medical technicians (EMTs). For these participants, the experience of administering naloxone added a human dimension to their role, creating opportunities for empathy and connection with those they might have otherwise viewed only through the lens of criminal behavior.

“Just like how police officers are expected to catch the bad guy, like EMTs are expected to save the person. Does it always happen? No. You try really hard on both aspects, but I feel like on one job it’s almost like the expected result, versus like they didn’t expect the cop to come in and, you know, have Narcan sitting in his pocket. It was cool. It was a cool experience.”

*(Participant BA09)*

While officers acknowledged that the expansion to a more public safety-oriented role allowed them to engage with community members in new ways, some noted, that this balance could be challenging, especially in situations where they might otherwise be required to enforce laws against drug use. For these participants, naloxone introduced a tension related to the dual role of law enforcer and life saver. As one officer highlighted:

“We follow what the department tells you to do. For example... even though he has a needle in his arm and he’s overdosed, we’re not going to arrest him... It’s not an arrest. Helping somebody that is overdosed or using drugs, that is still a crime.”

*(Participant RC07)*

Further, as officers adapted to their new role, some saw the provision of naloxone as extending beyond just the immediate overdose response, reflecting a more compassionate approach to policing. Despite mixed feelings about these responsibilities, the transition to administering naloxone highlighted the potential for law enforcement to play a pivotal role in harm reduction and community health.

“But carrying [naloxone], maybe they’ll look at us as we’re trying to help them more than trying to arrest them, which I guess is a good thing, especially if they’re on the borderline of overdosing. I would rather them try to welcome us showing up when they’re highly intoxicated. Once the drugs are in the system, some people don’t understand that the crime’s over. I can’t arrest you for that. You took it. Now I just want to make sure you live.”

*(Participant AH09)*

### Naloxone as a tool to soften public perceptions of police

Officers frequently discussed how administering naloxone could help improve the often adversarial relationship between the police and the public, particularly among people who use drugs. For some officers, carrying naloxone was seen as a way to highlight the police’s role in saving lives rather than enforcing laws or conducting arrests. One officer reflected on how he had garnered positive media coverage after administering naloxone in the field.

“They thought it was great. One of them actually has the news article hanging up in the office still, so they felt, hey it’s great the cops, you know, they didn’t throw this person in jail, but hey they saved the guy’s life. So it shows oh look they care and that we’re doing more than just standing there. So I think it was a little bit of positive.”

*(Participant AH04)*

Administering naloxone also afforded participants the chance to serve in a role that countered commonly held views about police work as largely punitive. Intervening in an overdose and saving a life enabled officers to actively address the overdose crisis and be an agent of change. This shift in role challenged some of the negative stereotypes of policing and gave participants a new way to connect with the public and an increased sense of value in their professional work.

“I feel better because... I realize right away what it is and I know, and I’m there... It makes me feel better. Because, like I said, the parents are there, they’re nice people. They’re calling us for help. We got to help. And I have the tool to help them so I got to do whatever I can.”

*(Participant RC06)*

Despite recounting positive interactions, there remained skepticism about the extent to which naloxone administration would alter entrenched negative views of the police. As some officers noted, while individual interactions might be positive, the broader public perception had not changed dramatically. As one officer reported, despite carrying a potentially life-saving tool, people continue to be apprehensive about the intent of the police as compared to EMS.

“I think they’re very afraid of us when we show up... The people don’t wanna open up to us... Like they have a bad persona, you know, maybe from like past police contact that they’ve been arrested before for possession or something and they think that, you know, because they still have like that bad image of us that they’re afraid to ask us for help as opposed to willingly speaking with EMS.”

*(Participant BA09)*

### Overdose reversal as facilitating drug use and crime

Officers described the frustration they experienced when reviving individuals who continued to use drugs despite repeated overdoses. Several officers voiced concern that naloxone might inadvertently enable further drug use, as individuals who survive an overdose may feel less deterred from engaging in risky behaviors. For example, one officer expressed frustration about repeatedly encountering the same individuals in overdose situations:

“It makes me sad when I have to go to the same location and the same person is overdosed again. That it hasn’t changed anything after the person gets, I mean that the person didn’t die because of the overdose and we use it and the person is still using drugs.”

*(Participant RC07)*

Likewise, several officers expressed that the immediate life-saving benefits of naloxone might unintentionally reinforce behaviors that they viewed as harmful or criminal. Officers voiced concerns that without complementary treatment or other types of intervention, naloxone could inadvertently function as a safety net that emboldened continued substance use without addressing its underlying causes.

“We should carry Narcan, but for these guys that are repeat offenders where they’re getting Narcan all the time, it’s a catch-22. It’s a great tool, but it’s also a hindrance, because some of these people think, hey, I can get high, I can almost die, and then my friend could shoot me up or I can even Narcan myself.”

*(Participant RC03)*

Reflecting on their peers, some participants reported that other officers in their precinct had commented that reversing overdoses might propagate drug markets to the benefit of people who sell drugs. This catalyzed a tension that, while naloxone might enable an officer to save a life in the immediate, if the person continued to use drugs, it could inadvertently end up supporting the illicit drug market.

Half of them think, “Okay, we’re doing good.” The other half are like, “What did you save? You know, you’re just making the drug dealer more rich.” I mean, they’ll save them, but... it’s like, “You just made a drug dealer rich. You’re just giving them money. It’s giving them money.””

*(Participant AH03)*

### People who use drugs as unworthy of revival

Several officers described an internal conflict they and their colleagues experienced regarding the “worthiness” of those they revived and reported a tension between their duty to save lives and their feelings about whether some individuals deserved to be saved. This was particularly salient when officers described reversing multiple overdoses for a single individual or when a reversal did not catalyze treatment or recovery for the person who overdosed, generating a perception that some people who use drugs may be beyond help.

“Honestly, I’m not the type to make that judgment call. Let God or whoever you believe does that do that. But do a lot of people think that, too? Yeah. This is a junkie. This is someone who’s been arrested 20 times. This is someone who knows better. This is someone who made that choice. This is something that a lot of cops think, absolutely. They think it’s a waste of paper and a waste of time because it’s going to happen again, and it, a lot of times, does happen again. So it’s kind of like... Why’d you save that junkie? Why did you do that? Look who it was.”

*(Participant RC03)*

For other officers, their frustrations with overdose reversal extended to questions of resource allocation and their capacity to “save lives” of different groups. For example, one officer highlighted the contrast between resources available for overdose interventions versus other emergencies:

“There is a little resentment that it’s like [overdose is] the easiest thing for me to fix that’s so life threatening. It’s like, you know, an EpiPen doesn’t even work that well and you’re telling me oh I give one dose of this stuff, maybe two, depending on how much of an addict they are they’re going to be perfectly fine. But the kid that accidently had a peanut butter and jelly sandwich, you know, he might die?... That’s my only like resentment, is like why is something so simple, it’s for the idiot that does it to himself.”

*(Participant AH04)*

Lastly, officers frequently expressed that overdose reversal should be coupled with treatment to interrupt patterns of substance use and help people before it was “too late.” These sentiments regarding coercive treatment were closely aligned with notions of worthiness described above. One officer expressed this succinctly:

“Just like on the repeat offender... like I really wish there was a way that was like mandatory rehab, you know, before you watch this person die. You know, there’s only so many times you can overdose and play around with it until you had a really bad batch or it’s too late”

*(Participant BA09)*

### Naloxone as a limited tool in the face of cross-system failures

While officers generally recognized naloxone as a valuable tool for saving lives, they also expressed frustration with its limitations within the broader context of systemic failures. Several officers pointed out that while naloxone could reverse an overdose, it does little to address the underlying issues of addiction and lacks the support needed for long-term recovery. One officer expressed frustration with the current approach to post-overdose care, noting that individuals are often released from the hospital with limited referrals to voluntary treatment services:

“You save them. You put them in the ambulance. They go to the ER. The doctor checks them out. And ‘Okay we suggest you go to rehab. And have a nice day.’”

*(Participant AH03)*

Officers also highlighted that their naloxone training was primarily procedural, limited in scope, and lacked a broader educational component on substance use disorders and associated services and treatments. These perceived training limitations further contributed to participants’ sense that they were unequipped to address the complexities of overdose cases beyond immediate overdose reversal. One officer suggested that the need for more comprehensive intervention strategies extended beyond policing and required cross-system coordination, as well as training should better explain what opioids are and how to recognize an opioid overdose, noting that uncertainty could make officers reluctant to administer naloxone when presentations might “mimic” other conditions:

“I think [overdose] should be better explained, like what an opiate is, as opposed, and what to look for as an opiate. I know the best thing about Narcan is that if you give it... So that’s one of the biggest things is that I think people are reluctant using it. Like, what happens if he’s a diabetic, or if it’s mimicking something else, and we give it, which I think that needs to be... emphasized a lot better than, you know, when in doubt just give it. And I think that, you know, it should be expressed that you will backed up for it... We just know drugs is drugs, you know. We don’t know exactly how they work on the human body as cops.”

*(Participant AH07)*

## Discussion

This study explored the experiences of naloxone administration to reverse opioid overdoses by NYPD officers in NYC. Findings illuminated the complexities and challenges faced in officers’ evolving roles, as public health approaches to the overdose epidemic are integrated into policing practice. We documented five key themes: (1) transitioning officer roles from law enforcement to public safety, (2) naloxone as a tool to soften public perceptions of police, (3) overdose reversal as facilitating drug use and crime, (4) people who use drugs as unworthy of revival, and (5) naloxone as a limited tool in the face of cross-system failures. Overall, findings offer insights into the perceptions and attitudes of police officers at the frontline of the overdose epidemic, with implications for public health and safety policy, police officer training, and interagency collaboration.

Findings highlighted that training on overdose response and furnishing officers with naloxone facilitated a transition from traditional law enforcement to more expansive “public safety” roles, which may reflect a broader paradigm shift in policing [56]. The sense of fulfillment in saving lives that officers in our study expressed aligns with prior research indicating that law enforcement personnel often find value in roles that emphasize community service and public health [57,58]. The adoption of naloxone equips officers with the means to provide an immediate and life-saving intervention, thus expanding their capacity to serve the community in a way beyond enforcement. However, the dual role of enforcer and caregiver raised challenges. That officers reported tensions in balancing their duty to uphold the law with the emergent responsibility to administer a healthcare intervention—especially in situations involving illegal drug use—echoed findings from previous studies that documented officer uncertainty about their role in health-related interventions and potential conflicts of interest with their enforcement duties [34,42]. Together, this suggests that clear guidelines and supportive policies are essential to navigate the complexities of integrating public health strategies into policing practice, ensuring officers are prepared and confident in their expanded roles.

While participants framed naloxone as a symbol of a shift from traditional enforcement toward broader public safety, this interpretation should be understood within a longer history of police responding to non-criminal crises. As a lineage of scholars have noted, police have long served as the de facto first responders to situations involving mental illness, substance use, and other social or health emergencies [59–61]. Naloxone does not uniquely mark a break with law enforcement agencies’ prior engagement with social and health crises, past but rather offers officers a contemporary, medically sanctioned tool to intervene directly in a life-threatening health crisis. In this sense, naloxone’s significance may lie less in creating a new role and more in highlighting the tensions inherent in officers’ existing hybrid functions at the intersection of enforcement, public safety, and care.

Crucially, the themes identified in this study are consistent with prior qualitative research in other jurisdictions. For example, studies from Canada, the United Kingdon, and elsewhere in the US have documented similar tensions in how officers reconcile their enforcement roles with emergent public health responsibilities [33,62–65]. This convergence across settings reinforces the need for upstream interventions, such as structural reform, stigma reduction training, and interagency alignment, rather than relying solely on street-level tools like naloxone. By adding NYPD officer perspectives, representative of a unique urban epicenter and the largest police department in the US [45], this study contributes to the growing evidence base highlighting the limitations of downstream public health interventions when implemented in law enforcement contexts.

The emergent themes related to stigma and the worth of people who use drugs may be interpreted within the broader established literature on how police occupational culture shapes moral evaluation of people who use drugs and, in turn, receptivity to harm reduction [37^,66].^ In our data, experiences of repeat overdose interventions functioned as explanatory frames for officer frustration and provided a mechanisms to reify stigmatizing beliefs that may erode procedural fairness and narrow officers’ views regarding the priority of coercion over care. Contemporary evidence suggests that officers’ support for harm reduction is patterned by underlying beliefs about addiction (e.g., chronic disease vs. moral failing) and by organizational cues that either legitimize referral-oriented practices or reinforce punishment defaults [33]. Advances in police-focused harm reduction training and guidance that explicitly pairs naloxone access with anti-stigma education, clear diversion or deflection protocols at overdose scenes, and credible pathways to voluntary treatment and harm reduction may therefore be necessary to prevent naloxone from becoming a technically effective but socially corrosive intervention [67].

Notably, a significant concern among officers in our study was that overdose reversal might inadvertently facilitate continued drug use and associated criminal activity. The perception that naloxone might enable risky behaviors reflects a pervasive public misunderstanding of harm reduction approaches: the assumption that interventions intended to save lives may inadvertently increase risk by removing deterrents to dangerous activities [68]. Similar sentiments have been documented in other studies, where first responders expressed frustration with repeat overdose cases and questioned the effectiveness of naloxone without adequate follow-up support [42,69]. Given the scope of police use of naloxone and persistent increases in overdose mortality in the US, it is crucial that public health and law enforcement officials address these concerns. Adequate and systematic education and training for officers that emphasize harm reduction principles and an understanding of substance use disorder as a chronic disease that police are positioned to “treat” through naloxone administration may help reframe and mitigate negative and stigmatizing officer sentiments [32,70].

Moreover, integrating police overdose responses with pathways to voluntary treatment, harm reduction, and social services through established partnerships with healthcare and social services—as has been done in other countries [71,72]—can enhance the effectiveness of interventions, increase trust between people with substance use disorders and the police, and alleviate officer perceptions of the limitations of naloxone alone. Other countries, such as Portugal and Canada [73,74], may offer templates for more integrated harm reduction policing models, through which officers may be co-located with health and social service providers or explicitly trained to support diversion to care rather than enforcement. These approaches reflect structural and cultural shifts that more fully embed harm reduction within public safety systems, and jurisdictions across the US have begun to integrate such approaches into practice. Likewise, engaging officers in the development and implementation of overdose response strategies as they continue to expand can enhance officer and community buy-in and address law enforcement reservations [75,76].

Although these interviews predate several major shifts in overdose policy and practice, they offer analytic value as an early implementation snapshot of how patrol officers interpreted the introduction of a lifesaving and, at the time novel, public health intervention into routine policing. In that early period, officers described tensions that are less about any single naloxone program configuration than about the durable organizational and cultural dilemmas that emerge when enforcement institutions are asked to deliver health-oriented responses: role ambiguity, stigma and deservingness judgments, and the limited impact of downstream tools when treatment and social service systems remain fragmented [77]. Accordingly, these findings should not be read as describing current NYPD practice, but rather as documenting an earlier phase of policy adoption that helps illuminate persistent implementation challenges, heightened in a contemporary policy landscape that emphasizes law enforcement over public safety [78]. Importantly, subsequent innovations—including expanded deflection and diversion infrastructures [79], evidence-based officer trainings that emphasize health integration (e.g., SHIELD) [67], and substantial federal investments in public health and public safety partnerships [80]—can be understood as attempts to operationalize solutions to precisely these recurrent tensions, suggesting that historical qualitative data can still inform the design and refinement of contemporary training, supervision, and cross-sector protocols.

Although the interviews in this study were conducted in 2016–2017, the policy landscape surrounding both naloxone access and police involvement in overdose response has evolved significantly since that time. Nationally, naloxone has become increasingly normalized in policing, with a 2025 national survey of police chiefs reporting that 84% of departments equip officers with naloxone [81]. In NYC, the landscape has shifted with the establishment of the first two publicly recognized overdose prevention centers in the US in 2021 [82], as well as the expansion of diversion and deflection programs aimed at reducing criminal legal consequences for people who use drugs [83]. Over-the-counter (OTC) approval of naloxone nasal spray by the US Food and Drug Administration in 2023 further broadened public access to naloxone [84]. These developments reflect a wider movement toward embedding harm reduction within public safety systems. Yet our findings remain salient: officers continue to occupy an ambiguous space between enforcement and care, and the challenges of stigma, fragmentation, and role conflict endure. Understanding the early experiences of officers navigating these tensions provides a valuable foundation for informing contemporary implementation strategies grounded in on-the-ground realities.

### Practice Implications

Findings from this study highlight several considerations for law enforcement agencies integrating overdose response into their public safety mission. First, officers may benefit from clear training and guidance on the role of naloxone within broader public health strategies, especially when navigating tensions between enforcement and care, exemplified by evidence-based training protocols such as Project SHIELD [85]. In particular, training may need to include practical decision-making support on overdose recognition and when to administer, including reassurance about the clinical safety profile of naloxone when overdose is suspected and EMS is en route, to reduce hesitation driven by diagnostic uncertainty [26]. Second, while naloxone can serve as a bridge to community trust, its effectiveness may depend on consistent messaging, visible accountability, and strong partnerships with health and social service providers, connections that may be built and solidified institutionally through public health and public safety partnerships [86]. Finally, officers may need organizational support to manage the emotional toll of repeated overdose encounters and to avoid reinforcing stigma toward PWUD and reigniting professional tensions between law enforcement and public health [87]. Agencies may invest in cross-sector collaboration, trauma-informed supervision, and data systems that align overdose response with upstream interventions [17]. Embedding these practices into police culture can strengthen both public health and public safety outcomes.

### Limitations

This study has several limitations. First, interviews were restricted to officers of the NYPD, the local police department with jurisdiction in NYC. Given the NYPD’s size, scope, and substantial investments in community policing, findings may not generalize to police departments in other jurisdictions. However, as the largest and most influential police force in the US, with an international reach, findings about NYPD officer experiences are likely to provide insight for practitioners and policymakers in other settings [45]. Second, most participants in the sample identified as white (87%) and male (73%). Given the importance of officers’ social identities in shaping their perspectives on policing, substance use, and community engagement, this demographic homogeneity may limit the breadth of insights captured. Future research should aim to recruit more demographically diverse samples, including officers of different races, ethnicities, and gender identities, to better understand how varied lived experience may influence attitudes toward overdose response and public health roles [88]. Third, this study was conducted prior to the widespread adoption and over-the-counter approval of intranasal naloxone in the US and, as such, findings were captured during a different naloxone policy climate [89]. At the time of data collection (2016–2017), NYPD officers carried intranasal naloxone hydrochloride delivered via a mucosal atomization device (MAD) that required manual assembly (i.e., a prefilled syringe plus a separate atomizer) [90], with a typical adult intranasal dose delivered via MAD of 2 mg/2 mL total (1 mg/1 mL in each nostril), with repeat dosing as clinically indicated [91]. However, given the focus on police officer experiences of administering naloxone in the course of duty, findings remain relevant for law enforcement practitioners regardless of the available naloxone formulations in a given country or region. Fourth, findings reflect self-reported officer experiences and thus may be subject to social desirability bias given the sensitive nature of the topics discussed [92]. However, assurances of confidentiality and considerations of respondent safety were prioritized by the research team to generate a safe environment for respondents [93]. Fifth, because interviews were conducted prior to the widespread emergence of myths about passive fentanyl exposure, our data do not capture how these beliefs may influence officers’ perceptions of overdose response or motivations for carrying naloxone, particularly the framing of naloxone as a tool for officer self-protection rather than community care [94]. Sixth, the formulation of naloxone carried by NYPD officers at the time of data collection (2016–2017) required manual assembly of an applicator device [95]. This delivery method was more cumbersome compared to the prefilled nasal spray cartridges now widely used by law enforcement nationally [96]. The effort and perceived complexity associated with assembling the device may have affected officers’ perceptions of usability and willingness to carry or deploy naloxone, an aspect on which our interviews did not elaborate, focused instead of officers’ experiences of reversal and social role. As such, findings on barriers to adoption should be interpreted in light of these now-outdated administration protocols. Seventh, the data reflect officer experiences and perceptions prior to several substantial shifts in the overdose epidemic, including the proliferation of fentanyl and polysubstance contamination [97], broad-based institutional acceptance of police access to naloxone [18], the maturation of public health and public safety partnerships [14], and development of integrated training protocols (e.g., SHIELD) [67]; as such, findings should be interpreted as a time-specific snapshot that may not fully represent contemporary practices or attitudes in NYPD or other jurisdictions.

### Conclusions

The integration of naloxone administration into police practice represents a significant shift in the role of law enforcement as the field adapts to address the overdose epidemic. Our findings demonstrate that while officers value the ability to save lives, they may also grapple with challenges related to their evolving responsibilities, public perceptions, and systemic limitations. Addressing these challenges requires comprehensive strategies that include policy clarity, enhanced training, interagency collaboration, and efforts to reduce stigma. By understanding and supporting officers in their expanded roles that incorporate public health, law enforcement agencies can more effectively contribute to overdose prevention and strengthen community relationships.

## References

[1] U.S. Overdose Deaths Decrease Almost 27% in 2024. Centers for Disease Control and Prevention. 2025. https://www.cdc.gov/nchs/pressroom/nchs_press_releases/2025/20250514.htm

[2] Centers for Disease Control and Prevention. Provisional Data Shows U.S. Drug Overdose Deaths Top 100,000 in 2022. National Center for Health Statistics, Centers for Disease Control and Prevention. 2023.

[3] Scholl L, Seth P, Kariisa M, Wilson N, Baldwin G. Drug and opioid-involved overdose deaths - United States, 2013-2017. MMWR Morb Mortal Wkly Rep. 2018;67(51–52):1419–27. 30605448 10.15585/mmwr.mm675152e1 PMC6334822

[4] Zoorob M. Fentanyl shock: The changing geography of overdose in the United States. Int J Drug Policy. 2019;70:40–6. doi: 10.1016/j.drugpo.2019.04.010 31079029

[5] Shover CL, Falasinnu TO, Dwyer CL, Santos NB, Cunningham NJ, Freedman RB, et al. Steep increases in fentanyl-related mortality west of the Mississippi River: Recent evidence from county and state surveillance. Drug Alcohol Depend. 2020;216:108314. doi: 10.1016/j.drugalcdep.2020.108314 33038637 PMC7521591

[6] Zibbell JE, Asher AK, Patel RC, Kupronis B, Iqbal K, Ward JW, et al. Increases in Acute Hepatitis C Virus Infection Related to a Growing Opioid Epidemic and Associated Injection Drug Use, United States, 2004 to 2014. Am J Public Health. 2018;108(2):175–81. doi: 10.2105/AJPH.2017.304132 29267061 PMC5846578

[7] Marks LR, Nolan NS, Liang SY, Durkin MJ, Weimer MB. Infectious Complications of Injection Drug Use. The Medical Clinics of North America. 2022;106(1):187–200. 34823730 10.1016/j.mcna.2021.08.006

[8] Cranston K, Alpren C, John B. Notes from the field: HIV diagnoses among persons who inject drugs - Northeastern Massachusetts, 2015-2018. MMWR Morb Mortal Wkly Rep. 2019;68(10):253–4. 30870405 10.15585/mmwr.mm6810a6 PMC6421964

[9] Peters PJ, Pontones P, Hoover KW. HIV infection linked to injection use of oxymorphone in Indiana, 2014-2015. The New England Journal of Medicine. 2016;375(3):229–39. 27468059 10.1056/NEJMoa1515195

[10] Lyss SB, Buchacz K, McClung RP, Asher A, Oster AM. Responding to outbreaks of human immunodeficiency virus among persons who inject drugs - United States, 2016-2019: perspectives on recent experience and lessons learned. The Journal of Infectious Diseases. 2020;222(Suppl 5):S239–49. 10.1093/infdis/jiaa112 32877545

[11] Brundage C, Fifield A, Partridge L. The Ripple Effect: National and State Estimates of the U.S. Opioid Epidemic’s Impact on Children. United Hospital Fund. 2020.

[12] Luo F, Li M, Florence C. State-Level Economic Costs of Opioid Use Disorder and Fatal Opioid Overdose - United States, 2017. MMWR Morb Mortal Wkly Rep. 2021;70(15):541–6. doi: 10.15585/mmwr.mm7015a1 33857070 PMC8344997

[13] Florence C, Luo F, Rice K. The economic burden of opioid use disorder and fatal opioid overdose in the United States, 2017. Drug Alcohol Depend. 2021;218:108350. doi: 10.1016/j.drugalcdep.2020.108350 33121867 PMC8091480

[14] Wolff J, Gitukui S, O’Brien M, Mital S, Noonan RK. The Overdose Response Strategy: Reducing Drug Overdose Deaths Through Strategic Partnership Between Public Health and Public Safety. J Public Health Manag Pract. 2022;28(Suppl 6):S359–66. doi: 10.1097/PHH.0000000000001580 36194807 PMC9531982

[15] White MD, Perrone D, Malm A, Watts S. Narcan cops: Officer perceptions of opioid use and willingness to carry naloxone. Journal of Criminal Justice. 2021;72:101778. doi: 10.1016/j.jcrimjus.2020.101778

[16] Mueller SR, Walley AY, Calcaterra SL, Glanz JM, Binswanger IA. A Review of Opioid Overdose Prevention and Naloxone Prescribing: Implications for Translating Community Programming Into Clinical Practice. Subst Abus. 2015;36(2):240–53. doi: 10.1080/08897077.2015.1010032 25774771 PMC4470731

[17] Allen B, Urmanche A. NYC RxStat: Stakeholder perspectives on a national model public health and public safety partnership to reduce overdose deaths. Eval Program Plann. 2023;98:102275. doi: 10.1016/j.evalprogplan.2023.102275 36924570 PMC10192163

[18] Ray B, Richardson NJ, Attaway PR, Smiley-McDonald HM, Davidson P, Kral AH. A national survey of law enforcement post-overdose response efforts. Am J Drug Alcohol Abuse. 2023;49(2):199–205. doi: 10.1080/00952990.2023.2169615 36820614

[19] Allen B, Feldman JM, Paone D. Public health and police: building ethical and equitable opioid responses. Proc Natl Acad Sci U S A. 2021;118(45). 10.1073/pnas.2118235118 PMC8609228 34732582

[20] Saucier CD, Zaller N, Macmadu A, Green TC. An Initial evaluation of law enforcement overdose training in Rhode Island. Drug Alcohol Depend. 2016;162:211–8. doi: 10.1016/j.drugalcdep.2016.03.011 27020323

[21] Dahlem CH, Patil R, Khadr L, Ploutz-Snyder RJ, Boyd CJ, Shuman CJ. Effectiveness of take ACTION online naloxone training for law enforcement officers. Health Justice. 2023;11(1):47. doi: 10.1186/s40352-023-00250-9 37979100 PMC10656891

[22] Dahlem CH, King L, Marr A, Holliday E. Web-Based Naloxone Training for Law Enforcement Officers: A Pilot Feasibility Study. Prog Community Health Partnersh. 2023;17(2):255–64. doi: 10.1353/cpr.2023.a900206 37462554

[23] Wagner KD, Bovet LJ, Haynes B, Joshua A, Davidson PJ. Training law enforcement to respond to opioid overdose with naloxone: Impact on knowledge, attitudes, and interactions with community members. Drug Alcohol Depend. 2016;165:22–8. doi: 10.1016/j.drugalcdep.2016.05.008 27262898

[24] Xavier J, Greer A, Crabtree A, Ferencz S, Buxton JA. Police officers’ knowledge, understanding and implementation of the Good Samaritan Drug Overdose Act in BC, Canada. Int J Drug Policy. 2021;97:103410. doi: 10.1016/j.drugpo.2021.103410 34438275

[25] Richardson NJ, Ray B, Smiley-McDonald HM, Davis CS, Kral AH. National survey findings on law enforcement agency drug response practices, overdose victim outcomes, and Good Samaritan Laws. Drug Alcohol Depend. 2023;248:109916. doi: 10.1016/j.drugalcdep.2023.109916 37236060

[26] Banta-Green CJ, Beletsky L, Schoeppe JA, Coffin PO, Kuszler PC. Police officers’ and paramedics’ experiences with overdose and their knowledge and opinions of Washington State’s drug overdose-naloxone-Good Samaritan law. J Urban Health. 2013;90(6):1102–11. doi: 10.1007/s11524-013-9814-y 23900788 PMC3853169

[27] Davis CS, Johnston J, de Saxe Zerden L, Clark K, Castillo T, Childs R. Attitudes of North Carolina law enforcement officers toward syringe decriminalization. Drug Alcohol Depend. 2014;144:265–9. doi: 10.1016/j.drugalcdep.2014.08.007 25193720 PMC4428167

[28] Olgin GK, Bórquez A, Baker P, Clairgue E, Morales M, Bañuelos A, et al. Preferences and acceptability of law enforcement initiated referrals for people who inject drugs: a mixed methods analysis. Subst Abuse Treat Prev Policy. 2020;15(1):75. doi: 10.1186/s13011-020-00319-w 33008431 PMC7530855

[29] Smiley-McDonald HM, Attaway PR, Richardson NJ, Davidson PJ, Kral AH. Perspectives from law enforcement officers who respond to overdose calls for service and administer naloxone. Health Justice. 2022;10(1):9. doi: 10.1186/s40352-022-00172-y 35212812 PMC8874742

[30] Dahlem CH, Granner J, Boyd C. Law Enforcement Perceptions About Naloxone Training and Its Effects Post-Overdose Reversal. J Addict Nurs. 2022;33(2):E2. 10.1097/JAN.0000000000000467 PMC9175640 35640219

[31] Pourtaher E, Payne ER, Fera N, Rowe K, Leung S-YJ, Stancliff S, et al. Naloxone administration by law enforcement officers in New York State (2015-2020). Harm Reduct J. 2022;19(1):102. doi: 10.1186/s12954-022-00682-w 36123614 PMC9483860

[32] Davis CS, Carr D, Southwell JK, Beletsky L. Engaging Law Enforcement in Overdose Reversal Initiatives: Authorization and Liability for Naloxone Administration. Am J Public Health. 2015;105(8):1530–7. doi: 10.2105/AJPH.2015.302638 26066921 PMC4504282

[33] Green TC, Zaller N, Palacios WR, Bowman SE, Ray M, Heimer R, et al. Law enforcement attitudes toward overdose prevention and response. Drug Alcohol Depend. 2013;133(2):677–84. doi: 10.1016/j.drugalcdep.2013.08.018 24051061 PMC3947507

[34] Carroll JJ, Mital S, Wolff J, Noonan RK, Martinez P, Podolsky MC, et al. Knowledge, preparedness, and compassion fatigue among law enforcement officers who respond to opioid overdose. Drug Alcohol Depend. 2020;217:108257. doi: 10.1016/j.drugalcdep.2020.108257 32947173 PMC7475730

[35] Winograd RP, Stringfellow EJ, Phillips SK, Wood CA. Some law enforcement officers’ negative attitudes toward overdose victims are exacerbated following overdose education training. Am J Drug Alcohol Abuse. 2020;46(5):577–88. doi: 10.1080/00952990.2020.1793159 32931324

[36] Del Pozo B, Reichert J, Martins K, Taylor B. Police Use of Discretion in Encounters with People with Opioid Use Disorder: a Study of Illinois Police Officers. J Police Crim Psychol. 2024;39(1):141–56. doi: 10.1007/s11896-023-09628-9 38617402 PMC11008765

[37] Reichert J, Del Pozo B, Taylor B. Police Stigma toward People with Opioid Use Disorder: A Study of Illinois Officers. Subst Use Misuse. 2023;58(12):1493–504. doi: 10.1080/10826084.2023.2227698 37365862 PMC10529704

[38] Reichert J, Martins KF, Taylor B, del Pozo B. Police knowledge, attitudes, and beliefs about opioid addiction treatment and harm reduction: A survey of Illinois officers. Journal of Drug Issues. 0(0):00220426231212567. doi: 10.1177/00220426231212567 PMC12094098 40406645

[39] Purviance D, Ray B, Tracy A, Southard E. Law enforcement attitudes towards naloxone following opioid overdose training. Subst Abus. 2017;38(2):177–82. doi: 10.1080/08897077.2016.1219439 27715714

[40] Jorgensen C. Badges and bongs: Police officers’ attitudes toward drugs. Sage Open. 2018;8(4):2158244018805357. doi: 10.1177/2158244018805357

[41] Murphy J, Russell B. Police Officers’ addiction frameworks and policy attitudes. Addict Behav. 2021;122:107007. doi: 10.1016/j.addbeh.2021.107007 34146796

[42] Lloyd D, Rowe K, Leung S-YJ, Pourtaher E, Gelberg K. “It’s just another tool on my toolbelt”: New York state law enforcement officer experiences administering naloxone. Harm Reduct J. 2023;20(1):29. doi: 10.1186/s12954-023-00748-3 36879248 PMC9987370

[43] van Dijk AJ, Herrington V, Crofts N, Breunig R, Burris S, Sullivan H, et al. Law enforcement and public health: recognition and enhancement of joined-up solutions. Lancet. 2019;393(10168):287–94. doi: 10.1016/S0140-6736(18)32839-3 30663598

[44] Delaney-Brumsey A. NYPD use of anti-overdose drug saves lives and recognizes substance use as public health issue. Vera Institute of Justice. 2014.

[45] Johnson BD, Golub A, McCabe J. The international implications of quality-of-life policing as practiced in New York city. Police Pract Res. 2010;11(1):17–29. doi: 10.1080/15614260802586368 20368765 PMC2847857

[46] Nguyen H, Parker BR. Assessing the effectiveness of New York’s 911 Good Samaritan Law-Evidence from a natural experiment. Int J Drug Policy. 2018;58:149–56. doi: 10.1016/j.drugpo.2018.05.013 29966919

[47] Bohler RM, Freeman PR, Villani J, Hunt T, Linas BS, Walley AY, et al. The policy landscape for naloxone distribution in four states highly impacted by fatal opioid overdoses. Drug Alcohol Depend Rep. 2023;6:100126. doi: 10.1016/j.dadr.2022.100126 36643788 PMC9838196

[48] Urmanche AA, Harocopos A. Experiences Administering Naloxone Among People in Different Social Roles: People Who Use Opioids and Family Members and Friends. J Drug Issues. 2023;53(3):475–89. doi: 10.1177/00220426221133024 37829614 PMC10569559

[49] Tong A, Sainsbury P, Craig J. Consolidated criteria for reporting qualitative research (COREQ): a 32-item checklist for interviews and focus groups. Int J Qual Health Care. 2007;19(6):349–57. doi: 10.1093/intqhc/mzm042 17872937

[50] Braun V, Clarke V. Using thematic analysis in psychology. Qualitative Research in Psychology. 2006;3(2):77–101. doi: 10.1191/1478088706qp063oa

[51] Guest G, MacQueen K, Namey E. Applied thematic analysis. SAGE Publications, Inc. 2012.

[52] Sandelowski M. Real qualitative researchers do not count: the use of numbers in qualitative research. Res Nurs Health. 2001;24(3):230–40. doi: 10.1002/nur.1025 11526621

[53] Del Pozo B, Magee L, Whiteside A, Thompson E, Martins K. The responsible conduct of police participatory research: A qualitative study of officers’ ethical beliefs. Res Ethics. 2025;:10.1177/17470161251349607. doi: 10.1177/17470161251349607 40881357 PMC12382591

[54] Stockdale KJ. Insider? Outsider? Reflections on Navigating Positionality When Researching Restorative Justice Policing. In: Armstrong S, Blaustein J, Henry A. Reflexivity and Criminal Justice: Intersections of Policy, Practice and Research. Palgrave Macmillan UK. 2017. 315–33.

[55] Weaver CM, Chu JP, Lugo A, Uyeda N, Cha Y, Zadonowicz T, et al. Community-based participatory research with police: Development of a tech-enhanced structured suicide risk assessment and communication smartphone application. Law Hum Behav. 2021;45(5):456–67. doi: 10.1037/lhb0000470 34871017

[56] Shepherd JP, Sumner SA. Policing and Public Health-Strategies for Collaboration. JAMA. 2017;317(15):1525–6. doi: 10.1001/jama.2017.1854 28358946 PMC5814117

[57] Watson AC, Wood JD. Everyday police work during mental health encounters: A study of call resolutions in Chicago and their implications for diversion. Behav Sci Law. 2017;35(5–6):442–55. doi: 10.1002/bsl.2324 29159822 PMC6295210

[58] Punch M, James S. Researching law enforcement and public health. Policing and Society. 2016;27(3):251–60. doi: 10.1080/10439463.2016.1205066

[59] Vitale AS. The end of policing. Verso. 2017.

[60] Bittner E. The Police on Skid-Row: A Study of Peace Keeping. American Sociological Review. 1967;32(5):699. doi: 10.2307/2092019

[61] Crawford A. Vulnerability and Policing: Rethinking the Role and Limits of the Police. Political Quarterly. 2024;95(3):431–41. doi: 10.1111/1467-923x.13422

[62] Speakman EM, Hillen P, Heyman I, Murray J, Dougall N, Aston EV, et al. “I’m not going to leave someone to die”: carriage of naloxone by police in Scotland within a public health framework: a qualitative study of acceptability and experiences. Harm Reduct J. 2023;20(1):20. doi: 10.1186/s12954-023-00750-9 36805681 PMC9938955

[63] Smiley-McDonald HM, Attaway PR, Richardson NJ, Davidson PJ, Kral AH. Perspectives from law enforcement officers who respond to overdose calls for service and administer naloxone. Health Justice. 2022;10(1):9. doi: 10.1186/s40352-022-00172-y 35212812 PMC8874742

[64] Xavier J, Greer A, Crabtree A, Buxton JA. Police officers’ perceptions of their role at overdose events: a qualitative study. Drugs: Education, Prevention and Policy. 2022;30(4):361–72. doi: 10.1080/09687637.2022.2070057

[65] Pike E, Tillson M, Staton M, Webster JM. The role of law enforcement officers in responding to the opioid epidemic: A qualitative assessment. Subst Abus. 2021;42(4):813–20. doi: 10.1080/08897077.2020.1865243 33471613 PMC8447229

[66] Trombley C, El-Shahawy O, Frank D. Once you’re labeled a drug user, you might as well stay the f*** home: Adverse police experiences among people who inject drugs. Subst Use Misuse. 2025;:1–9. 10.1080/10826084.2025.2588639 41340430

[67] Siddiqui ST, La Manna A, Connors E, Smith R, Vance K, Budesa Z, et al. An evaluation of first responders’ intention to refer to post-overdose services following SHIELD training. Harm Reduct J. 2024;21(1):39. doi: 10.1186/s12954-024-00957-4 38351046 PMC10863209

[68] Greene J. Naloxone “Moral Hazard” Debate Pits Economists Against Physicians. Annals of Emergency Medicine. 2018;72(2):A13–6. doi: 10.1016/j.annemergmed.2018.05.020

[69] Berardi L, Bucerius S, Haggerty KD, Krahn H. Narcan and Narcan’t: Implementation factors influencing police officer use of Narcan. Soc Sci Med. 2021;270:113669. doi: 10.1016/j.socscimed.2021.113669 33445119

[70] Baker P, Beletsky L, Garfein R, Pitpitan E, Oren E, Strathdee SA, et al. Impact of SHIELD Police Training on Knowledge of Syringe Possession Laws and Related Arrests in Tijuana, Mexico. Am J Public Health. 2022;112(6):860–4. doi: 10.2105/AJPH.2021.306702 35446602 PMC9137025

[71] McNeilly R, Berardi L, Haggerty KD, Bucerius SM, Krahn H. ‘Valuable Lives to Save’ vs. ‘Babysitting These People While They Try to Kill Themselves’: Changing Police Attitudes Towards Safe Consumption Sites. The British Journal of Criminology. 2024;64(6):1275–91. doi: 10.1093/bjc/azae021

[72] Sestoft D, Hansen SM, Christensen AB. The police, social services, and psychiatry (PSP) cooperation as a platform for dealing with concerns of radicalization. Int Rev Psychiatry. 2017;29(4):350–4. doi: 10.1080/09540261.2017.1343526 28805120

[73] Rêgo X, Oliveira MJ, Lameira C, Cruz OS. 20 years of Portuguese drug policy - developments, challenges and the quest for human rights. Subst Abuse Treat Prev Policy. 2021;16(1):59. doi: 10.1186/s13011-021-00394-7 34273972 PMC8285857

[74] Jozaghi E, Bird L. A Plea for Harm Reduction Policing Involving People Who Use Drugs. Int J Health Policy Manag. 2018;7(8):766–7. doi: 10.15171/ijhpm.2018.29 30078300 PMC6077278

[75] Reichert J, Adams S, Taylor J, Del Pozo B. Guiding officers to deflect citizens to treatment: an examination of police department policies in Illinois. Health Justice. 2023;11(1):7. doi: 10.1186/s40352-023-00207-y 36750519 PMC9906953

[76] Signori R, Heinrich DP, Wootton AB, Davey CL. Relational continuity in community policing: Insights from a human-centred design perspective. Policing: A Journal of Policy and Practice. 2023;17. doi: 10.1093/police/paad038

[77] Del Pozo B, Sightes E, Goulka J, Ray B, Wood CA, Siddiqui S, et al. Police discretion in encounters with people who use drugs: operationalizing the theory of planned behavior. Harm Reduct J. 2021;18(1):132. doi: 10.1186/s12954-021-00583-4 34915910 PMC8675297

[78] Barocas JA. The Erosion of Harm Reduction. New England Journal of Medicine. 2025;393(19):1865–7. 41211927 10.1056/NEJMp2511727

[79] Blais E, Brisson J, Gagnon F, Lemay S-A. Diverting people who use drugs from the criminal justice system: A systematic review of police-based diversion measures. Int J Drug Policy. 2022;105:103697. doi: 10.1016/j.drugpo.2022.103697 35489210

[80] Solomon AL. Public Health and Public Safety Partnerships: Charting a Response to the Overdose Epidemic. J Public Health Manag Pract. 2022;28(Suppl 6):S275–6. doi: 10.1097/PHH.0000000000001638 36194793 PMC9531969

[81] Bailey A, Andraka-Christou B, Rouhani S, Clark MH, Atkins D, Del Pozo B. Beliefs of US chiefs of police about substance use disorder, fentanyl exposure, overdose response, and use of discretion: results from a national survey. Health Justice. 2025;13(1):13. doi: 10.1186/s40352-025-00318-8 40042571 PMC11881252

[82] Harocopos A, Gibson BE, Saha N, McRae MT, See K, Rivera S, et al. First 2 Months of Operation at First Publicly Recognized Overdose Prevention Centers in US. JAMA Netw Open. 2022;5(7):e2222149. doi: 10.1001/jamanetworkopen.2022.22149 35838672 PMC9287749

[83] Labriola MM, Peterson S, Taylor J. A multi-site evaluation of law enforcement deflection in the United States. RAND Corporation. 2023.

[84] Rangachari P, Tran A. Transforming Opioid Overdose Prevention in the United States: Leveraging FDA’s Narcan Approval to Foster a Culture of Health. Risk Manag Healthc Policy. 2025;18:1935–46. doi: 10.2147/RMHP.S521720 40535536 PMC12176100

[85] Winograd R, Marotta PL, O’Neil MM, Siddiqui S, Connors E, La Manna A, et al. Improving first responders’ perceptions of overdose events and survivors through tailored occupational health-focused training co-facilitated by overdose survivors. Health Justice. 2024;12(1):49. doi: 10.1186/s40352-024-00309-1 39699777 PMC11660981

[86] Mital S, Wisdom AC, Wolff JG. Improving Partnerships Between Public Health and Public Safety to Reduce Overdose Deaths: An Inventory From the CDC Overdose Data to Action Funding Initiative. J Public Health Manag Pract. 2022;28(Suppl 6):S279–85. doi: 10.1097/PHH.0000000000001637 36194795 PMC9531979

[87] Goulka J, Del Pozo B, Beletsky L. From public safety to public health: Re-envisioning the goals and methods of policing. J Community Saf Well Being. 2021;6(1):22–7. doi: 10.35502/jcswb.184 34660886 PMC8516139

[88] Leon-Moreta A, Fayemiro O. Social and political pillars of police diversity: An institutional exploration. Urban Affairs Review. 0(0):10780874251364205.

[89] Zhu DT, Tamang S, Humphreys K. Promises and perils of the FDA’s over-the-counter naloxone reclassification. Lancet Reg Health Am. 2023;23:100518. doi: 10.1016/j.lana.2023.100518 37497396 PMC10366474

[90] Finger J. NYPD to be equipped with naloxone kits to fight heroin overdose. CBS News. 2014. https://www.cbsnews.com/news/nypd-to-be-equipped-with-naloxone-kits-to-fight-heroin-overdose/

[91] Regional Emergency Medical Services Council of New York City. Revision/Update of REMAC Prehospital Treatment & Transport Protocols. NYC REMAC. 2017.

[92] Habersaat S, Abdellaoui SH, Wolf JM. Social desirability, stress and health in police officers: preliminary results. PIJPSM. 2021;44(2):213–29. doi: 10.1108/pijpsm-08-2020-0133

[93] Bergen N, Labonté R. “Everything Is Perfect, and We Have No Problems”: Detecting and Limiting Social Desirability Bias in Qualitative Research. Qual Health Res. 2020;30(5):783–92. doi: 10.1177/1049732319889354 31830860

[94] Del Pozo B, Rich JD, Carroll JJ. Reports of accidental fentanyl overdose among police in the field: Toward correcting a harmful culture-bound syndrome. Int J Drug Policy. 2022;100:103520. doi: 10.1016/j.drugpo.2021.103520 34785420 PMC8810663

[95] Pourtaher E, Payne ER, Fera N, Rowe K, Leung S-YJ, Stancliff S, et al. Naloxone administration by law enforcement officers in New York State (2015-2020). Harm Reduct J. 2022;19(1):102. doi: 10.1186/s12954-022-00682-w 36123614 PMC9483860

[96] Bernosky-Smith K, Painter O, Butler S, Patel D, Clemency B, Lynch J. Intranasal overdose reversal formulations: a rapid review of available agents. Pain Manag. 2025;15(2):105–13. doi: 10.1080/17581869.2025.2461445 39902734 PMC11853544

[97] Ciccarone D. The rise of illicit fentanyls, stimulants and the fourth wave of the opioid overdose crisis. Curr Opin Psychiatry. 2021;34(4):344–50. doi: 10.1097/YCO.0000000000000717 33965972 PMC8154745

